# Pooled Specimen Testing Using Automated Cartridge-Based System for COVID-19: The Cost on Sensitivity

**DOI:** 10.21315/mjms2021.28.6.10

**Published:** 2021-12-22

**Authors:** Reem Y ALJINDAN, Amani M ALNIMR, Reem A AL DOSSARY, Ali J AL HADDAD, Fatimah A ALTURKI, Nouf Mohammed AL-ROMIHI, Bashayer Hussain ALDOSSARY, Khaled R ALKHARSAH

**Affiliations:** 1 Department of Microbiology, College of Medicine, Imam Abdulrahman Bin Faisal University (IAU), Dammam, Saudi Arabia; 2 Microbiology Laboratory, Medical Laboratory Department, King Fahd Hospital of the University, Al Khobar, Saudi Arabia

**Keywords:** SARS-CoV-2, COVID-19, pooling, Saudi Arabia, RT-PCR

## Abstract

**Background:**

Pooled specimen screening for the severe acute respiratory syndrome coronavirus 2 (SARS-CoV-2) can improve laboratory testing capacity. This study assessed the impact of pooling and retesting individual swabs on the overall detection rate and reduction in the frequency of retesting.

**Methods:**

One hundred respiratory swabs specimens were tested individually and in pools of three or five samples using the Cepheid’s Xpert^®^ Xpress SARS-CoV-2 test kit. The optimum number of samples per pool was calculated using the application ‘A Shiny App for Pooled Testing’.

**Results:**

Twenty-five pools were generated from 101 samples. Out of 13 pools that contained five samples each, three pools gave true positive results. While out of the 12 pools that contained three samples each, five pools gave true positive results. Four samples gave a false negative pool result. The overall sensitivity and specificity of the assay in the pools were 66.6% and 100%, respectively. The cycle threshold was reduced in most of the pools compared to individual sample tests.

**Conclusion:**

The overall pooled test had a remarkable impact on laboratory resources. Yet, caution is warranted when selecting the cases for pooled testing, since the reduction in sensitivity can significantly impact and increase the risk of exposure to infection.

## Introduction

The severe acute respiratory syndrome coronavirus 2 (SARS-CoV-2) pandemic instigated a huge impact on clinical microbiology laboratories and contributed to major challenges in the laboratory diagnosis. Considering the high infectivity of SARS-CoV-2, there is an urgent need for a rapid and accurate diagnostic assay for both clinical decisions and infection control purposes. Therefore, reverse-transcription polymerase chain reaction (RT-PCR) is considered the method of choice for diagnosing SARS-CoV-2 infection (1).

Despite the commercial availability of these tests, testing load is unprecedented and remains greater than the laboratory personnel, materials and equipment resources. In order to conserve the resources and overcome their ongoing shortage, several diagnostic laboratories considered pooled testing strategy that was accepted later on by the US Food and Drug Administration (FDA) for diagnostic, screening and surveillance purposes (2). At the same time, the results of pooled testing should maintain the reliability. Pooling strategy was introduced to laboratory testing long time ago and has been widely applied for human immunodeficiency virus (HIV) surveillance and blood donation, hepatitis C virus (HCV) and for some parasitic infections too (3). Pooling test for SARS-CoV-2 means running single molecular test for a mixture of respiratory specimens collected from different patients. Accordingly, if the result of a pooled test is negative, it would imply that all specimens that were included in the test are negative. On the other hand, a positive pooled test means that at least one of the included specimens is positive. Thus, individual testing is required for all specimens that were included in the positive pooled result. According to the FDA recommendations, the number of samples included in the pooling test should be optimised based on the prevalence of the disease in the community and the analytical sensitivity. The prevalence can be estimated by the rate of positivity of the virus within the last 7–10 days.

Generally, FDA stated that five is a reasonable specimen number for pooled testing for the initial validation process, given that the prevalence is 5%–6%. For populations with lower prevalence, a larger specimen number can be pooled. Although the pooled testing method appears to be cost-effective, it still has certain limitations. Hence, many important technical and clinical points need to be noted for pooled testing protocol. For example, pooling of different types of samples is not acceptable. Also, initial volume of the collected specimen needs to be sufficient enough for both pooled and individual testing. As pooling of samples is expected to reduce the analytical sensitivity, testing by pooling method needs to be clearly mentioned in the final laboratory report. Lastly, regular follow-up tests should be conducted to monitor the percentage of weak positive samples and the rate of positivity before and after applying the pooled testing strategy, while considering the possible delay in detecting a positive case (4).

To date, the available evidence for implementing pooled testing strategy is promising and suggests sufficient accuracy (5–8). To the best of our knowledge, the already published studies evaluated pooling strategy by different molecular system, but none used the fully automated molecular system. In this study, we aimed to evaluate the performance of pooled testing strategy using the Cepheid’s Xpert^®^ Xpress system.

## Methods

### Settings

This study was conducted prospectively at King Fahd Hospital of the University (KFHU), which is a 550-bed secondary health care institution in Al-Khobar Saudi Arabia, which receives referral of critically ill COVID-19 suspected patients from all the surrounding regions, as well as ambulatory cases.

### Specimens and Materials

The sample size was calculated as described previously (9). The following parameters were used: category 2 × 2 for contingency table; power at 90.0%; type I error of 0.05; κ2 (Cohen’s kappa [κ] coefficients for hypothesis testing) was set to 0.9, as the expected sensitivity of the assay is more than 90%; and the κ1 value was set to the values of 0.0, 0.3, 0.5 and 0.7. Considering these parameters, the estimated sample size ranged between 8 and 96 samples (9). One hundred and one samples were included in the study.

Combined nasopharyngeal (NP)/oropharyngeal (OP) swab specimens were submitted to the diagnostic microbiology laboratory at the hospital by the emergency department (ED) or employee health clinic for SARS-CoV-2 testing by RT-PCR. The swab specimens were transported in viral preservative medium (VPM) (Guangzhou Improve Medical Instruments, Guangzhou, China) refrigerated and processed within 24 h of collection. The Cepheid’s Xpert^®^ Xpress SARS-CoV-2 test kit (California, USA) was used to test all clinical specimens according to the manufacturer’s instructions. It is a cartridge-based system for the detection of the target sequence E and N2 gene of sarbecovirus and SARS-CoV-2, respectively. Residual sample volumes were stored at 8 °C after initial testing and then used in our pooling study within 24 h after collection.

### Inclusion and Exclusion Criteria

We included clinical samples obtained from the COVID-19 low-risk group (score below 5) based on COVID-19 clinical visual triage scoring. Samples were enrolled in the study in the order of their receipt in the laboratory to simulate a real-life scenario. Additionally, we included health care workers following a known and definite but unprotected exposure and patients tested as routine screening upon admission and prior to any surgical or radiological procedure. We excluded the repeated samples obtained from the laboratory-confirmed COVID-19 patients from the study.

### Specimen Pools

**Figure 1:**
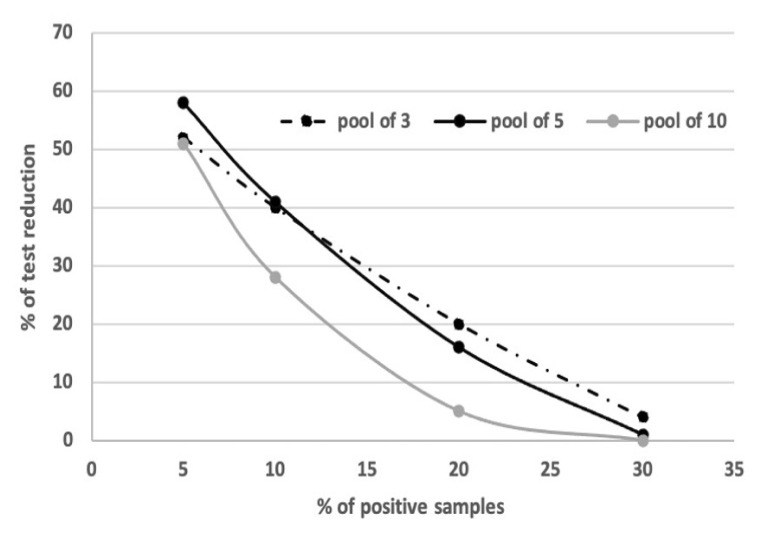
Percentage of reduction in the number of tests upon pooling of samples with respect to the percentage of total positive samples. Calculated using the application available at https://bilder.shinyapps.io/PooledTesting/, assuming that the sensitivity of the assay is 95% and the specificity of the assay is 99%

To calculate the optimum number of samples per pool and the percentage of test reduction while pooling the samples, we used the application ‘A Shiny App for Pooled Testing’ available at https://bilder.shinyapps.io/PooledTesting/, assuming that the sensitivity of the assay is 95% and its specificity is 99% (Figure 1). We estimated the percentage of positive SARS-CoV-2 tests (corresponding to disease prevalence in the application) to be 10%–12% based on the frequency of the positive samples in the two weeks preceding the study. Based on these circumstances, we chose to pool samples in pools of five and three, as it would lead to at least 40% reduction in the number of tests (Figure 1).

We used the residual verapamil from 101 previously tested NP/OP samples to create two groups of pools. The first group comprised pools of five samples per pool (65 samples into 13 pools). The second group comprised three samples per pool (36 samples into 12 pools). The samples were pooled in the order of their receipt in the microbiology laboratory. One hundred microlitres from each sample were taken for corresponding pool. Finally, 300 μL from the total quantity of the pool were taken for the RT-PCR test. The pools were tested as soon as they were compiled.

## Data Analysis

The results were tabulated in Excel spreadsheets. The sensitivity, specificity, negative likelihood ratio, negative predictive value, and accuracy were calculated using the MedCalc website (https://www.medcalc.org/calc/diagnostic_test.php). The κ coefficient was calculated using the κ free calculator website (https://idostatistics.com/cohen-kappa-freecalculator/). The PCR results of pooled samples were compared to the initial result of each individual sample. The results of the tested pool were considered true positive when the RT-PCR result of the pool was positive and there were one or more positive individual samples within the same pool. When both pooled samples and all of its compound samples were negative, the pool was considered as true negative. If the result of pooled samples was negative, and there was a positive sample on individual sample testing, the result was considered a false negative. The changes in the cycle threshold (Ct) values of E and N2 genes between the pooled sample and positive individual tests were also evaluated.

## Results

Out of 13 pools that contained five samples each, three pools gave true positive results and eight pools gave true negative results, which was in agreement with the test results of individual samples (Table 1). On the other hand, out of the 12 pools that contained three samples each, five pools gave true positive results and six pools gave true negative results, which was again in agreement with the test results of individual samples (Table 1). The percentage of false negative pool results was less among three-sample pools (Table 1). The agreement between the two testing strategies was significant (percentage of agreement: 96.04%; κ: 0.78) based on the data shown in Table 2. Compared to individual sample testing, the sensitivity of the pooling strategy was 66.67% (34.89%–90.08%) and its specificity was 100% (95.94%–100%) (Table 2).

The Ct value was higher for the pooled samples than for the individual sample tests, except for two pools (pool number 16 and 21) for which the Ct value for the pooled samples was lower than that for the corresponding individual samples (Table 3).

## Discussion

Due to the lack of adequate reagent supply for SARS-CoV-2 testing, several studies have suggested implementation of the strategy of pooling samples to scale up testing throughput and efficiency and to overcome the reagent shortage (5, 10–12). Several models have been designed to estimate the optimum size of sample groups (13). Sample groups of 3–11 have been found to be optimal, and even groups of 32 samples have been suggested to be possible, however, only for low-risk patients (6, 14). With a disease prevalence of approximately 10%, as reflected by the number of positive cases from the total tested samples, we estimated that a pool size of three to five samples would result in about 40% reduction in tests, and therefore, total cost.

Our data showed that pooling of samples for SARS-CoV-2 testing markedly reduced the number of conducted tests and, therefore, increased the testing capacity of the clinical microbiology laboratory. To avoid obtaining high positivity rate, we carefully selected the cases to have low pre-test probability score based on the visual triage assessment protocol followed at our hospital. A score of four or less indicated that a patient had a contact with confirmed COVID-19 case or worked in a facility or lived in an area known to be undergoing an outbreak of COVID 19 in the 14 days preceding to the onset of symptoms and exhibited only one of the symptoms that are not typical for COVID-19, such as headache, nausea, vomiting or diarrhea. Indeed, several other studies suggested employing pooling strategy for screening of patients who have a low risk of infection (5, 6, 15).

Our data also showed that pooling of samples markedly and negatively affects the sensitivity of the assay. The Cepheid’s Xpert^®^ Xpress SARS-CoV-2 test kit has been reported to be highly sensitive compared to other commercially available and laboratory-based assays (16–18). Hence, the compromised sensitivity with pools of samples is more likely attributed to dilution of the low viral load-positive sample in the pool. This was also evident by the observed increase in the Ct values of most of the positive sample for both targets in the assay upon pooling. Some studies have also reported a reduction in the sensitivity of the assay in the pooled samples; however, most of the studies have shown a minor effect of pooling on the sensitivity (8, 14, 19). In the current study, three samples (4.6%) exhibited a false negative result in the group of five samples per pool, while only one sample (2.8%) exhibited a false negative result in the group of three samples per pool. This outcome additionally emphasises on the effect of target dilution after pooling, since less number of false negative results were obtained after pooling three samples compared to after pooling five samples. According to the records at the Saudi Ministry of Health (MOH), the sample with pool ID number 7 (Table 3) was tested positive for SARS-CoV-2 with another assay 21 days before testing at our facility. This sample gave a positive SARS-CoV-2 test result on individual testing and false negative when tested in the pool. Despite the possibility that we might have detected a remnant nucleic acid in this sample rather than an infectious virus, this finding indicated the high sensitivity of the Cepheid’s Xpert^®^ Xpress SARS-CoV-2 test kit and that pooling will definitely reduce the analytical sensitivity of the assay. The sample with pool ID number 18 (Table 3) recorded negative result with a different assay at the Saudi MOH laboratories one day after the test result. This sample had a low viral load as evident by the Ct value and could have been misdiagnosed by the assay used at the Saudi MOH as indicated in our previous study that compared our assay to other molecular assays (data not shown). No record at the Saudi MOH was found for the rest of samples with false negative pool results. The four samples were asymptomatic and attended the hospital seeking health care for other medical conditions. Bearing in mind the high infectivity of SARS-CoV-2, failing to promptly and properly diagnose a case with SARS-CoV-2 infection will expose a large number of individuals who may develop COVID-19, including medical staff and housemates who would have the highest risk of being infected. Previous reports have documented the consequences of misdiagnosing a patient with SARS-CoV-2 infection in multiple COVID-19 local outbreaks (20–22).

Two of samples in this study exhibited a lower Ct value in the pool than in the single test. Such effect has been previously reported and was attributed to a carrier effect of multiple positive samples in the pool (23). In our study, these two samples were the only positive samples in the pool and this effect, therefore, could be attributed to a reaction inhibitor that was diluted in the pool. However, this observation needs to be studied further.

The limitations of our study include the small sample size and the selection of swab specimens. Further, larger studies need to address the potential use and cost-effectiveness of specimen pooling in certain patient populations with low risk and optimise the pool size and sample volume accordingly. Additionally, the clinical team needs to be aware that a pooling strategy is being implemented in the laboratory, which may reduce assay sensitivity, especially with large pool sizes.

## Conclusion

In this study, we examined the effect of pooled specimen testing for COVID-19 using a fully automated nucleic acid amplification test. The overall pooled test accuracy and diagnostic performance had a remarkable effect on laboratory resources. Yet, caution is warranted while selecting the cases for pooled testing, since the reduction in sensitivity can have significant impact if the misdiagnosed cases result in high-risk infectiousness circumstances, such as in pre-surgical patients.

## Figures and Tables

**Table 1:** Summary of the COVID-19 sample pool-results and percentage of test reduction

	Pools			Total	% Test reduction*

	Positive	Negative	False negative		
Pool of 5	3	8	2	13	53
Pool of 3	5	6	1	12	21

Note:

* Calculated for samples and pools with agreement

**Table 2:** Evaluation of the diagnostic value of pooling COVID-19 samples for all pools in relation to individual testing

		Single sample		Total

		Positive	Negative	
Pooled sample	Positive	8	0	8
	Negative	4	89	93

	Total	12	89	101

Sensitivity			66.67%	
Specificity			100%	
Negative likelihood ratio			0.33	
Negative predictive value			95.70%	
Accuracy			96.04%	
% of agreement			96.04	
κ			0.78	

**Table 3:** Comparison of cycles threshold between the original and pooled COVID-19 samples

Pool ID	Samples/pool	Sample result		Pool result	

		Ct (E)	Ct (N2)	Ct (E)	Ct (N2)
1	5	37.2	39.6	44.1	41
3	5	32.4	34.8	33.7	36.5
6	5	37.4	36.8	0	0
7	5	37.4	40	0	0
7	5	0	40.4	0	0
10	5	35.1	37.1	39.3	42.3
16	3	36.3	39	34.3	37.1
18	3	0	41.8	0	0
20	3	32.5	35.1	33.8	36.9
21	3	36.3	39	32.7	35.7
23	3	22.6	24.6	23.4	25.4
24	3	36.9	39.6	39.4	42.2
